# Phosphatidic acid supplementation increases skeletal muscle hypertrophy and strength

**DOI:** 10.1186/1550-2783-10-S1-P13

**Published:** 2013-12-06

**Authors:** Jordan M Joy, Ryan P Lowery, Joshua E Dudeck, Eduardo O De Souza, Ralf Jäger, Sean A McCleary, Stephanie M C Wilson, Martin Purpura, Jacob M Wilson

**Affiliations:** 1 Department of Health Sciences and Human Performance, The University of Tampa, Tampa, FL, USA; 2 Laboratory of Neuromuscular Adaptations to Strength Training, School of Physical Education and Sport, University of São Paulo, São Paulo, Brazil (EDS; 3 Increnovo LLC, 2138 E Lafayette Pl, Milwaukee, WI, USA; 4 Department of Nutrition, IMG Performance Institute, IMG Academy, Bradenton, FL, USA

## Introduction

The accretion of skeletal muscle tissue can be critical for a varied population including athletes and elderly. Skeletal muscle hypertrophy is largely mediated through increased muscle protein synthesis. The mammalian target of rapamycin (mTOR) has been shown to regulate rates of muscle protein synthesis and a mechanical stimulus (resistance exercise) has been shown to activate mTOR with the phospholipid Phosphatidic Acid (PA) playing a key role. A first pilot study found that oral supplementation with soy-derived PA in athletes undergoing progressive resistance training very likely resulted in greater increases in squat strength and lean mass over the placebo. However, this pilot study was likely underpowered, the workout was not supervised and no direct measures of skeletal muscle hypertrophy were taken. Therefore, the purpose of this study was to investigate the effects of PA on body composition, strength, power and muscular hypertrophy.

## Methods

Twenty-eight resistance trained, male subjects (21 ± 3 years of age, bodyweight of 76 ± 9 kg, and height of 176 cm ± 9 cm) participated in this study. Subjects were equally divided into experimental and control conditions, and each subject took part in an 8 week periodized resistance training program. The resistance training program consisted of two hypertrophy oriented workouts per week and one strength oriented workout per week. The experimental condition (EXP) received 750 mg of soy-derived PA (Mediator™, Chemi Nutra, White Bear Lake, MN), while the control condition (CON) received a visually identical placebo (rice flour). Measurements of DEXA-determined body composition, rectus femoris CSA, 1RM strength, and anaerobic power were taken prior to and following the 8 week training intervention. A 2x2 repeated measures ANOVA was used to determine group, time, and group x time interactions. A Tukey post-hoc was used to locate differences.

## Results

There was a significant group x time effect (p=0.02) for CSA, in which the EXP group increased (+1.01 cm^2^, ES = 0.92) to a greater extent than the CON group (+0.61 cm^2^, ES = 0.52). There was a significant group x time effect (p=0.01) for LBM, in which the EXP group (+2.4 kg, ES = 0.42) doubled the effects of resistance training alone (CON +1.2 kg, ES = 0.26). There was a significant group x time effect (p=0.04) for leg press 1RM, in which the EXP group increased to a greater extent (+52.0 kg, ES = 1.2) than the CON group (+32.5 kg, ES = 0.78). There was a trend group x time effect (p=0.06) for fat loss, in which the EXP group decreased body fat to a greater extent than the CON group (-1.3kg vs. -0.5kg).

## Conclusion

Supplementation with soy-derived PA can improve responses in skeletal muscle hypertrophy, lean body mass, and maximal strength.

